# Modeling the Onset of Symptoms of COVID-19

**DOI:** 10.3389/fpubh.2020.00473

**Published:** 2020-08-13

**Authors:** Joseph R. Larsen, Margaret R. Martin, John D. Martin, Peter Kuhn, James B. Hicks

**Affiliations:** ^1^Quantitative and Computational Biology, Department of Biological Science, University of Southern California, Los Angeles, CA, United States; ^2^USC Michelson Center for Convergent Bioscience, University of Southern California, Los Angeles, CA, United States; ^3^Nexus Development PA LLC, Redwood City, CA, United States; ^4^NanoCarrier Co., Ltd., Chiba, Japan

**Keywords:** COVID-19, Markov, probability, symptoms, stochastic, model, disease, influenza

## Abstract

COVID-19 is a pandemic viral disease with catastrophic global impact. This disease is more contagious than influenza such that cluster outbreaks occur frequently. If patients with symptoms quickly underwent testing and contact tracing, these outbreaks could be contained. Unfortunately, COVID-19 patients have symptoms similar to other common illnesses. Here, we hypothesize the order of symptom occurrence could help patients and medical professionals more quickly distinguish COVID-19 from other respiratory diseases, yet such essential information is largely unavailable. To this end, we apply a Markov Process to a graded partially ordered set based on clinical observations of COVID-19 cases to ascertain the most likely order of discernible symptoms (i.e., fever, cough, nausea/vomiting, and diarrhea) in COVID-19 patients. We then compared the progression of these symptoms in COVID-19 to other respiratory diseases, such as influenza, SARS, and MERS, to observe if the diseases present differently. Our model predicts that influenza initiates with cough, whereas COVID-19 like other coronavirus-related diseases initiates with fever. However, COVID-19 differs from SARS and MERS in the order of gastrointestinal symptoms. Our results support the notion that fever should be used to screen for entry into facilities as regions begin to reopen after the outbreak of Spring 2020. Additionally, our findings suggest that good clinical practice should involve recording the order of symptom occurrence in COVID-19 and other diseases. If such a systemic clinical practice had been standard since ancient diseases, perhaps the transition from local outbreak to pandemic could have been avoided.

## Introduction

The current pandemic of Coronavirus Disease 2019 (COVID-19), caused by severe acute respiratory syndrome coronavirus 2 (SARS-CoV-2), has undergone an observed exponential increase of cases that has overrun hospitals across the world (1). Many people have mild forms of the disease and are advised not to go to the hospital or to seek a diagnostic test because they can recover at home. A large number of others are asymptomatic (2). Infected individuals are highly contagious and can transmit the disease even if they are asymptomatic, and this fact furthers the need to isolate and test often (2). In addition, COVID-19 is two to three times more contagious than influenza (3). Due to these characteristics, outbreaks of COVID-19 occur in clusters (4). Identifying COVID-19 early could reduce the number and size of clusters, but early symptoms are not well-defined. The Center for Disease Control and Prevention (CDC) in the USA and the World Health Organization (WHO) currently advise the public to call their doctor if they believe they have been exposed to COVID-19 or exhibit fever and cough (5). However, fever and cough are associated with other respiratory diseases such as influenza (6–8). Influenza, with an estimated number of symptomatic cases in the millions annually in the U.S. alone (9), also is commonly associated with fever and cough (6). Similarly to COVID-19, the Middle East Respiratory Syndrome (MERS) and the Severe Acute Respiratory Syndrome (SARS) are respiratory illnesses contracted from coronaviruses called the MERS-Related Coronavirus (MERS-CoV) and SARS-Related Coronavirus (SARS-CoV), respectively (7). The symptoms of these diseases also overlap with COVID-19. The capacity to discern differences in these common symptoms, such as order of occurrence and likely first symptoms, would aid in early recognition. If health care workers recorded and published clinically-observed and/or patient-reported sequences of symptoms, the reported data could be evaluated as an additional tool for early recognition of COVID-19 to increase self-surveillance and reduce spread. If such a widespread clinical practice had been instituted in the past, perhaps local outbreaks of influenzas, coronaviruses, and other diseases might have been contained before becoming pandemics.

To this end, we assumed that symptoms and their orders are independent variables and created a model that approximates the probability of symptoms occurring in specific orders using available, non-ordered patient data. The use of these assumptions and data was necessary given the lack of ordered data. To do this, we applied a Markov Process to determine the order of occurrence of common symptoms of respiratory diseases. We have previously used a Markov Chain to predict cancer metastasis location (10–14). A Markov Process is defined as a stochastic sequence of events in which the likelihood of the next state only depends on the current state rather than past or future states (15). In this case, we defined each state to be the specific symptoms that a patient has experienced, and each transition is only dependent on these symptoms. As a result, we can determine the likelihood of each symptom stepwise using a Markov Process. We defined the state probability of a node as the frequency that a patient has a particular combination of symptoms divided by the total number of patients that exhibit the same number of symptoms. The transition probability between two states is defined as the likelihood of acquiring a single specific symptom divided by the likelihood of acquiring all possible next symptoms. We then applied a greedy algorithmic approach using the transition probabilities to calculate the probability of all possible orders toward determining the most and least likely orders of symptoms.

In this study, we first defined this specific application of a Markov Process applied to a graded partially ordered set (poset), which we refer to as the Stochastic Progression Model. In this case, our graded poset represents all possible combinations of symptoms and all possible orders of symptom occurrence. It is graded because the possible combinations of symptoms are ranked by the number of symptoms that they each represent. For example, the symptom combination of fever and cough has the same rank as the combination of cough and diarrhea. We found that the Stochastic Progression Model for adults that are symptomatic indicates that there may be an order of discernible symptoms in COVID-19, but the order of symptoms seems to be independent of severity of the case on admission. From there, we compared the most likely order of symptoms in other respiratory diseases to COVID-19. To expand on our results, we analyzed a larger set of symptoms that are common to all respiratory diseases studied here and sought to decipher further distinctions.

## Materials and Methods

### Data Collection

Patient data from this study was collected from various reports in literature on the frequencies of symptoms in COVID-19, influenza, MERS, and SARS (Supplemental Tables 1, 2). Each dataset was used either to approximate order of symptoms, to confirm our results, or to analyze first symptoms in COVID-19 or influenza. For all of these applications, we used the reported patient data to simulate patients with various combinations of symptoms experienced and then applied the simulated data to perform the analyses.

The main dataset of COVID-19 patients of the World Health Organization, containing 55,924 confirmed cases, was obtained through review of national and local governmental reports and observations made during visits to areas with infected individuals in China that occurred from February 16 to 24, 2020 (8). A confirmation dataset of COVID-19 patients, containing 1,099 confirmed cases, was obtained by the China Medical Treatment Expert Group for COVID-19 from medical records and other compiled data of hospitalized patients and outpatients that were diagnosed with COVID-19. This data was reported to the National Health Commission of China from December 11, 2019 to January 29, 2020 (16). For both COVID-19 datasets, myalgia was reported as myalgia or arthralgia. We assumed that most patients with myalgia also had arthralgia, and therefore we used the frequency of myalgia or arthralgia as a frequency for myalgia when simulating data. The influenza dataset, containing 2,470 confirmed cases, was collected by researchers at the University of Michigan from a retrospective pooled analysis of mostly unvaccinated patients participating in phase 2 and 3 clinical trials that were conducted in North America, Europe, and the Southern Hemisphere from 1994 to 1998 (6). This group of patients has a mean age of 35 and each exhibited multiple symptoms. Vomiting and diarrhea were not reported in this influenza dataset, but they are common among respiratory disease. Although adult patients at times may experience vomiting and diarrhea when infected with influenza, these symptoms are rare (17). Therefore, we approximate the frequency of these symptoms as 0.010 in this case. The datasets representing symptom frequency in MERS, containing 245 patients, and SARS, containing 357 patients, were collected on admission and were reported as clinical data from physicians, Dr. Yin, at the Beijing Chao-Yang Hospital and Dr. Wunderink, at the Northwestern University Feinberg School of Medicine (7). The patients included in these datasets varied in age and pre-existing conditions. In the cases of SARS, the patients tended to be younger and have fewer pre-existing conditions than in the cases of MERS.

We used initial frequency data of MERS and SARS to further ascertain early symptoms of disease. The MERS initial symptom frequency dataset, containing 45 confirmed cases, was collected from electronic medical records at the Samsung Medical Center in Seoul, South Korea that contained onset symptom data about patients in the 2015 Korean MERS outbreak (Supplemental Table 3) (18). The SARS initial symptom frequency dataset, containing 144 confirmed cases, was collected from hospital records including information of early symptoms in patients dating from March 7 to April 10, 2003 during an outbreak in the greater Toronto area (Supplemental Table 4) (19).

Lastly, two additional datasets were collected to determine the utility of using first symptoms as early indicators of COVID-19 and influenza. The COVID-19 dataset used, containing 138 patients, was independent of all prior COVID-19 datasets. This data was obtained from electronic medical records of patients admitted to the Zhongnan Hospital of Wuhan University from January 1 to 28, 2020 (20). The symptom data was collected at onset of disease and all patients experienced pneumonia due to COVID-19. In this dataset, nausea and vomiting were reported separately for COVID-19. We assumed that most patients who experience vomiting, which is reported with a frequency of 0.036, also experience nausea, which is reported with a frequency of 0.101, and therefore to simulate the data, we defined the frequency for nausea/vomiting as 0.101. The influenza dataset used reported 20 confirmed cases of influenza and 400 confirmed negative cases of influenza and is independent from any other influenza dataset we used (21). The symptom data was collected through questionnaires and observations by medical professionals during the influenza seasons of 2006 and 2007 of infected patients admitted at the Department of Internal Medicine and Infectious Diseases and the Department of Pulmonology at the University Medical Center Utrecht. Like the other influenza dataset described above, vomiting and diarrhea were not reported in this dataset. So, we once again assumed the frequency of these symptoms to be 0.010 (17). Because this study was conducted in 2006 and 2007, prior to the COVID-19 outbreak, we assumed these patients were negative for COVID-19 as well. So, this 400-patient group was used as the dataset that represents individuals negative for both COVID-19 and influenza (Supplemental Table 5).

### Simulating Symptom Progression From Patient Data

The Stochastic Progression Model was built in R under version 3.5.2 and was illustrated by using the hasse function in the hasseDiagram_0.1.3 library (code available online: https://github.com/j-larsen/Stochastic_Progression_of_COVID-19_Symptoms) (22, 23). Each respiratory disease report was represented by a corresponding data frame, with columns as symptoms, one row as the frequency of the symptoms observed in the study, and the other row as the frequency multiplied by 1,000. The multiple of the frequency is defined as the frequency count, which represents the probability of a symptom in a theoretical sample size of 1,000 simulated patients. Additionally, the state of an individual is displayed through a character array of ones and zeros, where ones represent the presence of a symptom and zeroes represent its absence. This process of simulating a symptom is analogous to a jar of marbles of either two colors. The probability of pulling one color of marble (i.e., a specific symptom) is illustrated by the frequency count because the total number of marbles in the jar is 1,000 and the frequency count for each is the number of the specific color of marbles in the jar.

We then simulated data of 500,000 patients, by randomly selecting if a patient has or does not have a symptom using the procedure described above and storing that information in a data frame that represents patients as rows and symptoms as columns. We assumed the occurrence of symptoms are random and independent. Considering these assumptions, we built the character arrays by applying the jar of marbles method for each simulated patient. The method repeats for each patient and involves pulling a marble from a series of jars representing each symptom. The information from each randomly pulled marble is stored in the corresponding cell of the character array in the correct column representing the symptom and the row representing the simulated patient. This process is repeated for all 500,000 simulated patients for all symptoms.

### Building the Stochastic Progression Model

The Stochastic Progression Model is illustrated as a directed acyclic graph with nodes, representing the power set of Boolean vectors. The power sets of Boolean vectors each represent a possible state of a patient by noting the absence or presence of specific symptoms. The edges, which illustrate the transition from one state to another, were selected specifically using key definitions and assumptions to create a poset. We defined the states at the nodes as symptoms that a patient has experienced up until this point. We created and directed edges from states with fewer symptoms to more starting at the minimum set of a Boolean vector of all zeros, which indicates a person with no symptoms. First, we assume that each symptom occurs one at a time, even if the difference in time is infinitesimal. With this assumption, a node can only be directed to other nodes that denote the same set of symptoms plus one additional symptom. Second, we assume that if a patient does not digress and does not die, they will eventually acquire all symptoms reaching the maximum set of a Boolean vector, which represents a patient that has exhibited all symptoms. Applying these assumptions to form the directed acyclic graph creates a Hasse Diagram of a graded poset that follows a Markov Process altogether comprising the Stochastic Progression Model.

### Calculating State and Transition Probabilities

The nodes in the Hasse Diagram represent states of a patient by indicating the specific symptoms exhibited, and the edges represent transitions between these states. Therefore, we next needed to apply state probabilities to each node and transition probabilities to the directed edges. First, we labeled each simulated patient by summing the respective Boolean vector to find the number of symptoms for each patient. Then, to get the state probability of each node, we divided the number of simulated patients that are represented by the current Boolean vector by the total number of patients who have the same number of symptoms. To approximate the transition probability between two nodes (originating and terminating), we divided the number of simulated patients that are represented by the terminating node by the number of simulated patients that are represented by nodes characterized by the same number of symptoms as the terminating node, including the terminating node. The error of each node is determined by the sum of the products of the transition probabilities leading to that node subtracted from the state probability of the node. Then, the error of each implementation of the model was defined as the error of the node with the highest absolute value of error (Supplemental Figures 2–13). The transition probabilities signify the likelihoods of transitions from one node to another, and the aggregates of the transition probabilities in a sequence represent the likelihoods of the paths. These paths illustrate the order of symptoms when infected with a respiratory disease by observing the stepwise addition of symptoms when traversing down nodes in the path. The most and least likely paths were determined using a greedy algorithmic approach. This approach consists of selecting local maximum or minimum edges stepwise, which results in a most and least likely path, respectively. If the maximum (or minimum) transition probability from a specific node was within error of other transition probabilities of edges from the same originating node, we grouped the terminating nodes when finding the most (or least) likely path. In these cases, we could not distinguish a difference in likelihood between these specific transitions. The paths create a possible order of symptoms via the poset, each having a specific likelihood of occurrence.

## Results

### A Possible Order of Discernible Symptoms in COVID-19

**Figure 1:**
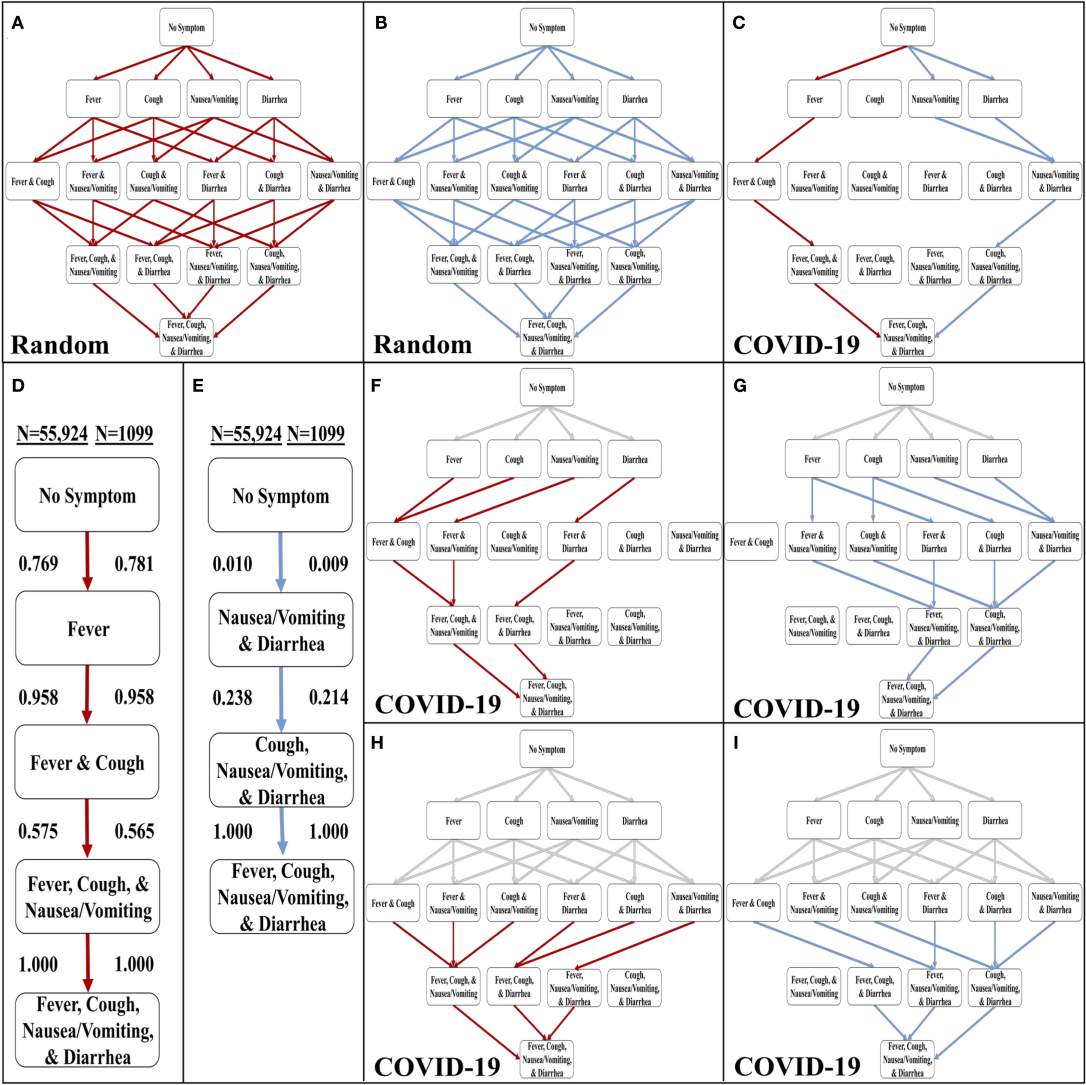
Development of the stochastic progression model for COVID-19. **(A)** The most likely paths (red) in the Hasse Diagram for symptoms with random likelihoods of occurring. **(B)** The least likely paths (blue) in the Hasse Diagram for symptoms with random likelihoods of occurring. **(C)** The most likely (red) and least likely (blue) paths in the Hasse Diagram for symptoms in COVID-19. **(D)** The most likely order of symptoms in COVID-19 based on our Stochastic Progression Model determined from transition probabilities presented here. **(E)** The least likely order of symptoms in COVID-19 based on our Stochastic Progression Model determined from transition probabilities presented here. **(F)** Hasse Diagram of the most likely paths (red) after traveling any forced path (gray) of patients with one symptom. **(G)** Hasse Diagram of the least likely paths (blue) after traveling any forced path (gray) of patients with one symptom. **(H)** Hasse Diagram of the most likely paths (red) after traveling any forced path (gray) of patients with two symptoms. **(I)** Hasse Diagram of the least likely paths (blue) after traveling any forced path (gray) of patients with two symptoms.

The WHO-China Joint Report from February 16 to 24, 2020 includes rates of symptom occurrence at presentation from 55,924 confirmed cases of COVID-19 (8). We identified symptoms that were easily discernible or objective (i.e., fever, cough, diarrhea, and nausea/vomiting) in comparison to other reported symptoms, such as inflammations of blood vessel epithelia (24), neurological effects (25), and rash-like symptoms (26). These symptoms are also common in other respiratory diseases. Thus, we chose to implement these four symptoms in the Stochastic Progression Model (Supplemental Table 1). To confirm the validity of the model, we first determined the possible sequences of symptom occurrence when the probabilities are uniformly random for each symptom. In addition to all possible orders of occurrence of the four symptoms, the diagram displays the most and least likely paths of the four symptoms, depicted by red lines and blue lines, respectively (Figures 1A,B). The most and least likely paths describe the most and least likely series of symptoms that a random infected person from the population in the dataset may experience. In this case, each possible path is equally likely, with no path having any higher probability than any other.

We then created another implementation of the Stochastic Progression Model and utilized the data in the WHO-China Joint Report (COVID-19 with *N* = 55,924) (8). With this implementation, we determined the most and least likely paths (Figure 1C). In this case, a person infected with COVID-19 is most likely to experience symptoms in the order of fever, cough, nausea/vomiting, then diarrhea (Figure 1D). The least likely path starts at diarrhea and nausea/vomiting and is followed by cough, and finally fever (Figure 1E). We confirmed these results with a smaller dataset (COVID-19 with *N* = 1,099) (Figures 1D,E, and Supplemental Figure 1) (16). The likelihoods of transitioning to fever, 0.769, and then to cough, 0.958, are high, and these observations indicate that a large portion of infected symptomatic patients may follow this path. Finally, this implementation of the model predicts that nausea/vomiting occurs before diarrhea. These two results suggest that in patients with SARS-CoV-2, the body first develops fever, then upper respiratory symptoms and finally symptoms of the upper then lower gastrointestinal (GI) tract.

To further investigate these symptom paths, we implemented the Stochastic Progression Model with the main dataset (COVID-19 with *N* = 55,924) (8), to determine the likely downstream paths when the first one or two symptoms are forced to a certain state (Figures 1F–I). The gray lines represent the “forced” paths. The rest of the paths were determined as before with a greedy algorithmic approach. We found that the most likely orders of the downstream path are consistent with the most likely orders of the unforced paths. Even if the first symptom is forced to be an unlikely one (e.g., diarrhea), the downstream paths maintain the most likely order of the other three symptoms that we originally determined (Figure 1F). Similarly, the GI tract effects occur first in the forced least likely paths (Figure 1G). When forcing the path one step further by predetermining the first two symptoms for both the most and least likely paths, the findings remain the same (Figures 1H,I).

### Order of Discernible Symptoms in COVID-19 Is Independent of Severity of Disease on Admission

**Figure 2:**
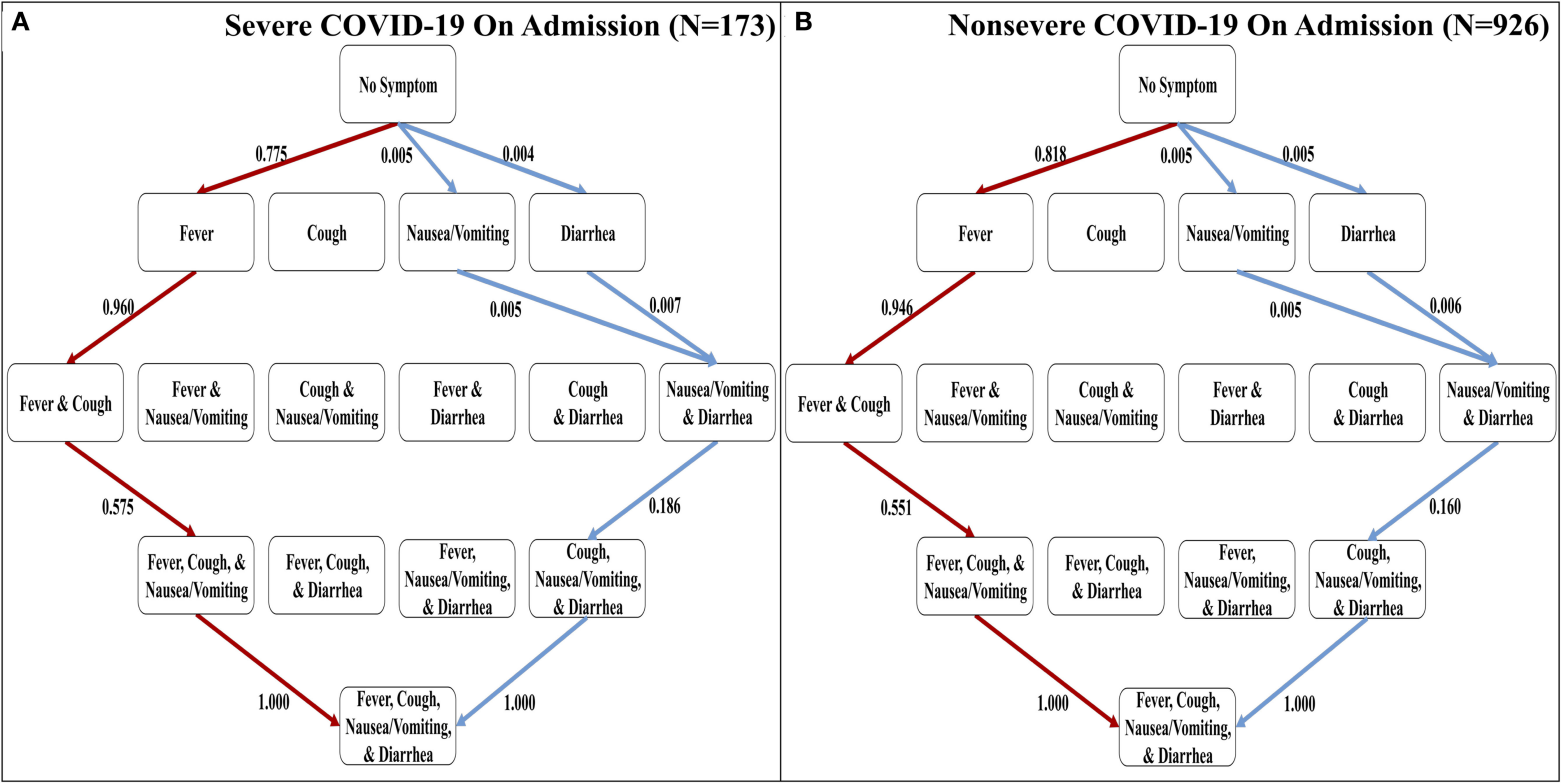
The most and least likely paths of discernible symptoms in severe and non-severe COVID-19 cases on admission. **(A)** Hasse Diagram of the most likely paths (red) and least likely paths (blue) in COVID-19 for cases designated as severe on admission determined from transition probabilities presented here. **(B)** Hasse Diagram of the most likely paths (red) and least likely paths (blue) in COVID-19 for cases designated as non-severe on admission determined from transition probabilities presented here.

The confirmation dataset of COVID-19 cases (*N* = 1,099) separates the reported 1,099 cases between severe and non-severe patients as designated on admission (16). To investigate the effects of severity on the order of discernible symptoms, we implemented each set of cases separately using the Stochastic Progression Model. We found that the most and least likely paths are identical in severe and non-severe cases and to our original findings above (Figure 2). To illustrate the similarities, the largest difference in likelihood is observed when transitioning from no symptoms to fever in the most likely path. In severe and non-severe cases, the probability is 0.775 and 0.818, respectively, indicating a difference of 0.043. These results suggest that severity does not affect the order of discernible symptoms, and they are consistent with the hypothesis of fever as the first symptom of COVID-19.

### Variation of Order of Discernible Symptoms Between Respiratory Diseases

**Figure 3:**
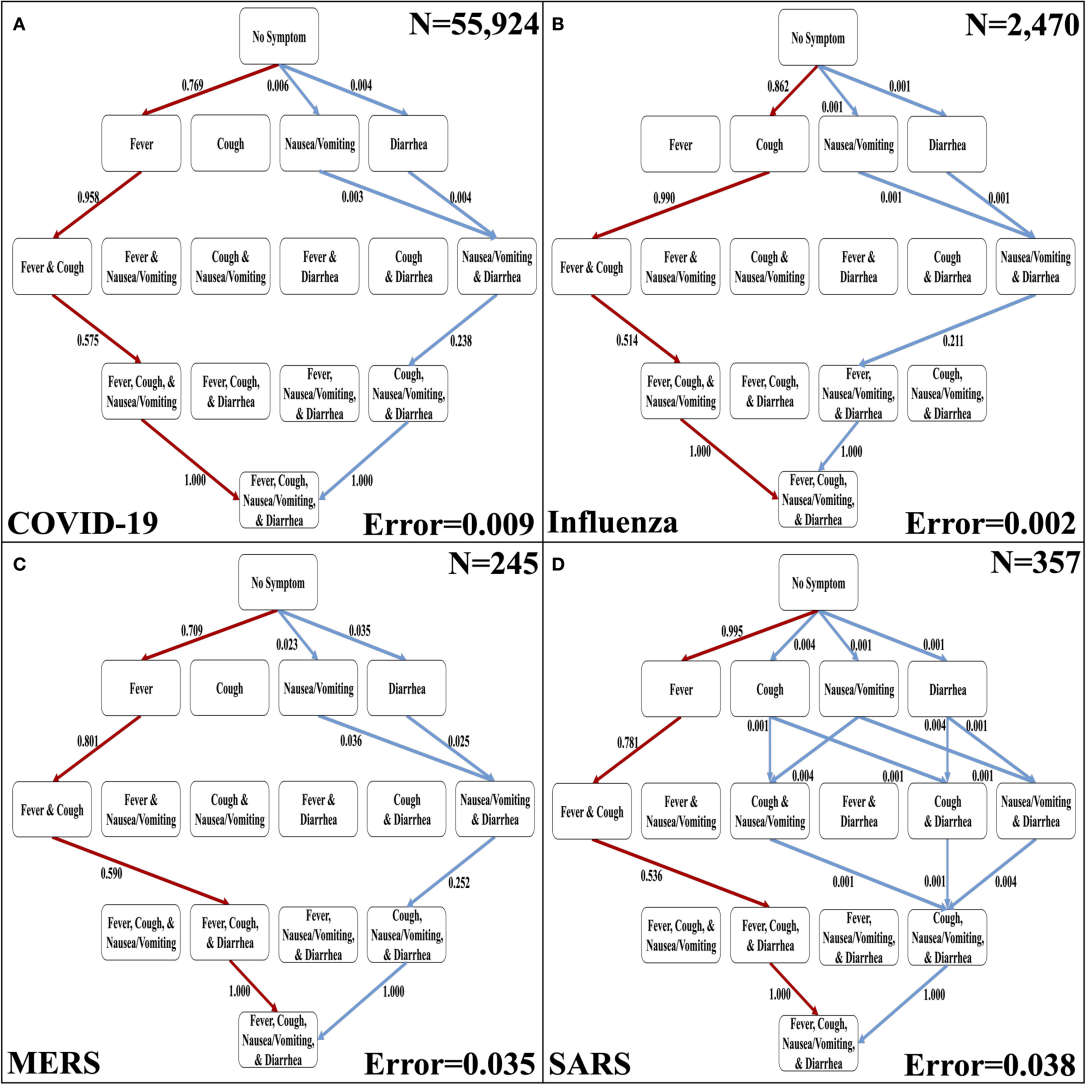
The most likely and least likely paths of discernible symptoms in respiratory diseases. **(A)** The most likely paths (red) and least likely paths (blue) in a Hasse Diagram for COVID-19 symptoms. **(B)** The most likely paths (red) and least likely paths (blue) in a Hasse Diagram for influenza symptoms. **(C)** The most likely paths (red) and least likely paths (blue) in a Hasse Diagram for MERS symptoms. **(D)** The most likely paths (red) and least likely paths (blue) in a Hasse Diagram for SARS symptoms. For each diagram, the most and least likely paths are determined from the transition probabilities that are depicted on the edges. Additionally, error of transition probabilities and sample size (*N*) are presented.

The four discernible symptoms are objective and relatively easy for patients and clinicians to confirm. So, we developed implementations of the Stochastic Progression Model using these symptoms to determine the most likely and least likely paths for four respiratory diseases: COVID-19, influenza, MERS, and SARS (Figures 3A–D) (6–8). The most likely order of occurrence of symptoms in COVID-19 is fever, cough, nausea/vomiting, and diarrhea (Figure 3A). This path is identical to influenza except the order of the initial two symptoms is switched (Figure 3B). On the other hand, the predicted most likely paths (i.e., fever, cough, diarrhea, and then nausea/vomiting) are the same for MERS and SARS (Figures 3C,D). This order has one difference from the most likely path in COVID-19 in that the order of the final two symptoms are reversed. The least likely path of MERS starts with either nausea/vomiting or diarrhea as the first step. These steps are followed by cough, and finally fever. In contrast, the least likely path of SARS is cough, nausea/vomiting, and diarrhea in any order, and then finally fever. However, the least likely path of symptoms in COVID-19 is the same as the least likely path in MERS, and the least likely path of influenza is unique compared to the other diseases. It is not detectable whether nausea/vomiting or diarrhea are the first symptoms in influenza, but after these two, the least likely path continues from there to fever then cough. This observation further illustrates the strong link of cough to influenza. As for coronavirus-related diseases, the strongest first indicator is fever followed by cough.

### Comparing the Order of Most Common Symptoms in Respiratory Diseases With COVID-19

**Figure 4:**
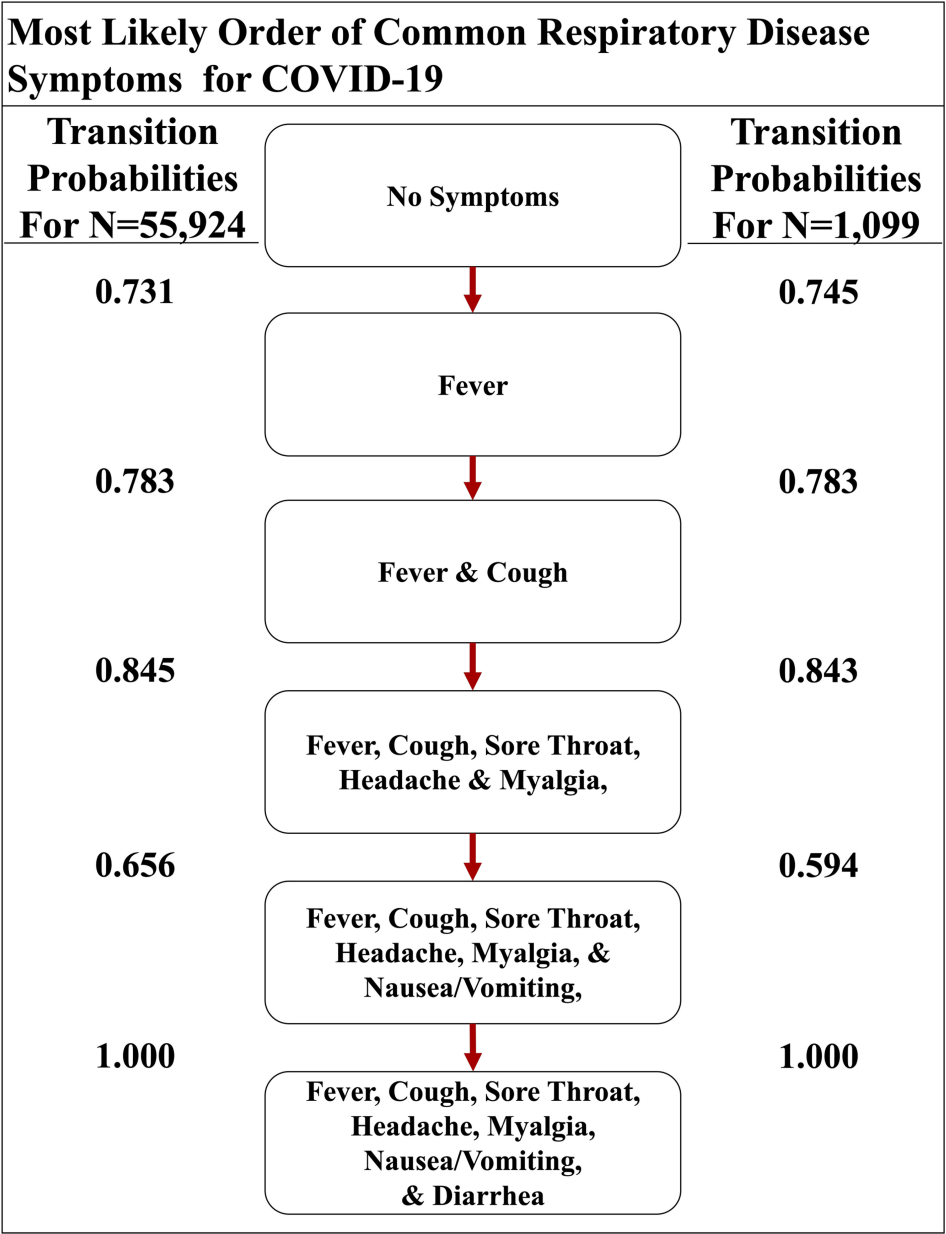
The most likely path of common respiratory symptoms in COVID-19. The most likely path of seven common symptoms of COVID-19, determined by the transition probabilities that are also listed between nodes, of two datasets here.

Although active surveillance of the order discernible symptoms (i.e., fever, cough, nausea/vomiting, and diarrhea) could be useful due to the distinctive most and least likely paths that we determined, we expanded our analysis to the seven symptoms commonly observed in all four respiratory diseases studied here. So, we created a second set of symptoms that amends sore throat, myalgia, and headache to the original set of symptoms (Supplemental Table 2). The three additional symptoms are more subjective (6–8). The seven-symptom implementation of the Stochastic Progression Model of COVID-19 shows that these additional symptoms did not perturb our initial ordering of fever, coughing, nausea/vomiting, and diarrhea, but instead added another level of intricacy in the middle of the likely paths (Figure 4). We still find that the most likely path first transitions to fever, indicating that fever is the most likely first symptom. From there, the most likely next symptom is cough once again. Then, we observe an undetectable difference in likelihood of transitioning to either sore throat, headache, or myalgia, indicating that all three are likely to occur next before proceeding. The final two nodes are consistent with the four-symptom order by indicating that nausea/vomiting then diarrhea occur last. Although this implementation is more complex because it has seven symptoms, it is consistent with our earlier findings. The most likely path of COVID-19 symptoms is fever, then cough, and next either sore throat, myalgia, or headache, followed by nausea/vomiting, and finally diarrhea, and this order is the same as the one indicated by the implementation developed from the confirmation dataset (COVID-19 with *N* = 1,099) (Figure 4) (16).

**Figure 5:**
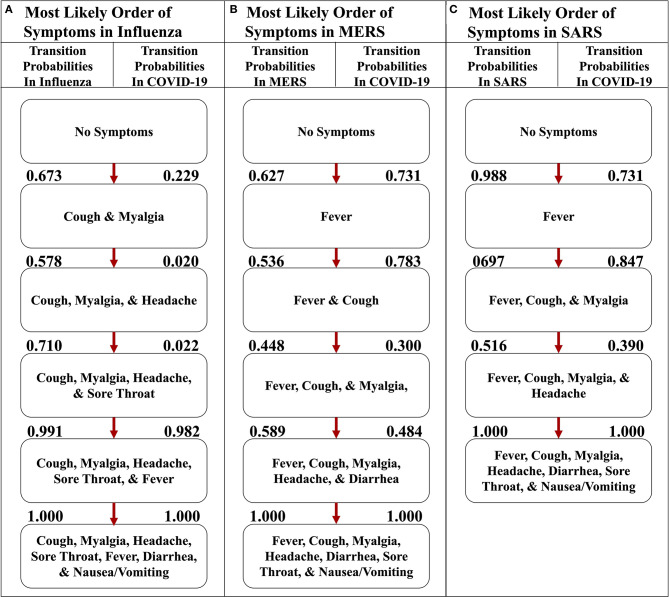
The most likely paths of symptoms in influenza, MERS, and SARS vs. COVID-19. **(A)** The most likely path of seven common symptoms of influenza with the transition probabilities listed between nodes. **(B)** The most likely path of seven common symptoms of MERS with the transition probabilities listed between nodes. **(C)** The most likely path of seven common symptoms of SARS with the transition probabilities listed between nodes. For each path, the transition probabilities in COVID-19 are listed on the right. The most likely paths for each respective disease here are determined from the transition probabilities listed between nodes on the left.

We also implemented the Stochastic Progression Model with the same seven symptoms in influenza, SARS, and MERS datasets to compare and contrast disease progression with that in COVID-19 (Figure 5) (6–8). The results for influenza indicate that cough or myalgia may occur first (Figure 5A). After these two symptoms occur, the order of symptoms is headache, sore throat and fever. Finally, vomiting/nausea and diarrhea have an undetectable difference in probability of occurring last. The MERS implementation displays a most likely path in which fever will occur first, followed by cough, headache, and then myalgia (Figure 5B). These are followed by an undetectable difference in likelihood of headache and diarrhea occurring. Finally, sore throat and nausea/vomiting will occur last with an undetectable difference. The implementation for SARS shows that fever is most likely to occur first, followed by an undetectable difference in transition probability of cough and myalgia, which is similar to the other coronavirus-related diseases (Figure 5C). Next, headache is most likely. Finally, diarrhea, sore throat and nausea/vomiting occur with an undetectable difference in likelihood.

**Figure 6:**
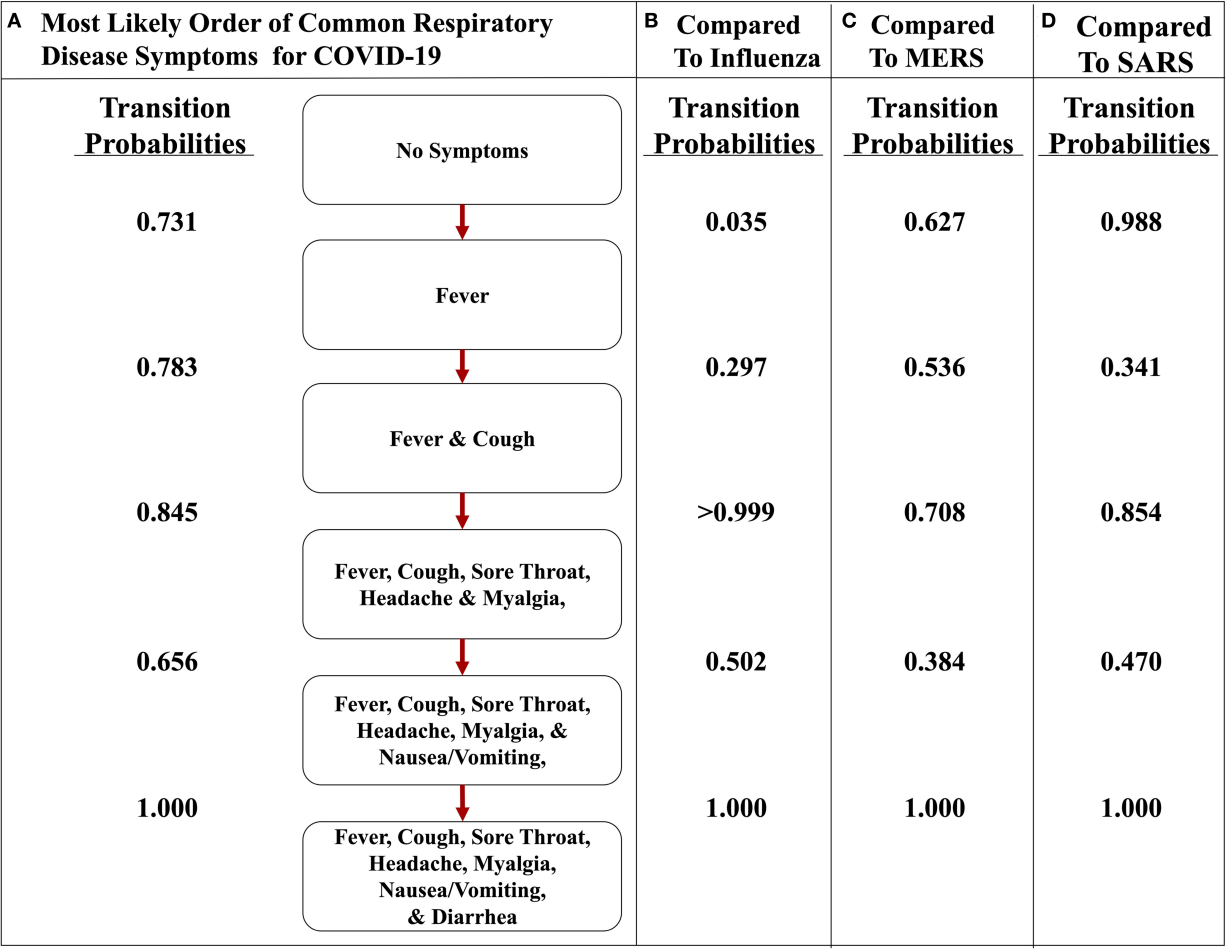
The most likely path of symptoms in COVID-19 vs. influenza, MERS, and SARS. **(A)** The most likely path of seven common symptoms of COVID-19 with the transition probabilities listed between nodes. **(B)** The transition probabilities of the path of influenza. **(C)** The transition probabilities of the path of MERS. **(D)** The transition probabilities of the path of SARS. The most likely path here is determined from the transition probabilities listed between nodes for COVID-19.

To illustrate the uniqueness of the most likely path of COVID-19, we found the transition probabilities of the same path in the other respiratory diseases (Figure 6). When comparing and contrasting the probabilities, we found that the implementation representing COVID-19 strongly asserts that the first symptom will be fever and cough will soon follow because the transition probabilities are 0.731 and 0.783, respectively (Figure 6A), whereas the influenza implementation indicates that fever is very unlikely to occur first with a probability of only 0.035 (Figure 6B). Additionally, the implementations of MERS and SARS data also have a high likelihood of transitioning to fever first, with a probability of 0.627 and 0.988, respectively (Figures 6C,D). The second symptom of the most likely path of COVID-19 is cough, with a probability of 0.783, but the others do not have a similar high probability. For example, the respiratory disease with the highest probability at that transition is MERS at 0.536. However, after fever and cough, COVID-19 and the other three respiratory diseases have a similarly high likelihood of the three subjective symptoms (i.e., sore throat, headache, and myalgia). Finally, the most likely path of COVID-19 ends with nausea/vomiting and then diarrhea. These observations are consistent with the symptoms described by the CDC and support the notion that fever followed by cough seems highly likely to be diagnosed as COVID-19 (5).

Also, comparing the transition probabilities of paths in the same disease illustrates the significance of the most likely pathways. For example, the lowest transition probability in the most likely path of influenza is 0.578 (Figure 5A), whereas when analyzing influenza as it traverses down the most likely path of COVID-19, the transition probabilities observed are 0.5 or less (Figure 6B). However, in that same path, the transition probability from fever and cough to fever, cough, sore throat, headache, and myalgia is >0.999. This value displays how unlikely nausea/vomiting and diarrhea are to be initial symptoms of influenza. Additionally, when observing the most likely path of COVID-19, the first two symptoms seem to have a strong probability of occurring in the order of fever and then cough, with a likelihood of 0.731 (Figure 5A). However, the likelihood of cough occurring first in COVID-19 is 0.229, which is a low probability (Figure 5A). This observation further supports the hypothesis of fever occurring first and cough occurring second.

### Recall and Selectivity When Linking First Symptom and Disease

The COVID-19 and influenza implementations of the Stochastic Progression Model suggest that there is a high likelihood of fever and cough occurring first, respectively. We desired to find metrics quantifying the possible link between first symptom and these two diseases. So, we determined the recall and the selectivity when using the initial symptom as an indicator of COVID-19 or influenza, with all other possible diseases excluded in a theoretical patient population. First, we simulated patient datasets using reported data that were independent from all previous work that we integrated in our analyses above (Supplemental Table 5) (20). Two simulated patient datasets were created to analyze COVID-19 and influenza separately to portray the specific link of each disease with the corresponding initial symptom that we determined, fever and cough, respectively. The simulated data contained information about the patients' state of disease (COVID-19, influenza or not) and their first symptom experienced. Based on the information of the first symptom alone, we categorized the simulated patient data as infected with COVID-19 or not and influenza or not. The recall was calculated as the number of simulated patients that we correctly identified as having the disease over the number of simulated patients that truly had the disease (27). Selectivity was defined here as the number of simulated patients that we correctly identified as not having the disease over the number of simulated patients that truly did not have the disease (28). For both diseases, we performed this analysis for five simulated samples of different sizes, each containing 5% infected individuals. We repeated this process 10 times and calculated the average and standard deviation across each sample size for both COVID-19 and influenza (Tables 1, 2).

**Table 1:** Recall and selectivity of linking fever as a first symptom of patients with COVID-19.

	**COVID-19**			
	**Recall**		**Selectivity**	
	**Mean**	**Standard deviation**	**Mean**	**Standard deviation**
10 Patients out of 200	0.980	0.063	0.661	0.030
20 Patients out of 400	0.990	0.021	0.665	0.030
30 Patients out of 600	0.977	0.035	0.668	0.017
40 Patients out of 800	0.973	0.018	0.665	0.020
50 Patients out of 1,000	0.966	0.031	0.665	0.016

*The mean and standard deviation of the recall and the selectivity of various simulations at different sample sizes that were each performed 10 times*.

**Table 2:** Recall and selectivity of linking cough as a first symptom of patients with influenza.

	**Influenza**			
	**Recall**		**Selectivity**	
	**Mean**	**Standard deviation**	**Mean**	**Standard deviation**
10 Patients out of 200	0.810	0.110	0.369	0.031
20 Patients out of 400	0.820	0.067	0.364	0.030
30 Patients out of 600	0.777	0.061	0.364	0.015
40 Patients out of 800	0.765	0.092	0.367	0.023
50 Patients out of 1,000	0.804	0.051	0.362	0.014

*The mean and standard deviation of the recall and the selectivity of various simulations at different sample sizes that were each performed 10 times*.

The recall ranges from 0.966 to 0.990 with a standard deviation of 0.031 and 0.021, respectively when analyzing the link between COVID-19 and fever as a first symptom. The maximum standard deviation of any sample size is 0.063 for the mean of 0.980. On the other hand, the selectivity of fever as a first symptom of COVID-19 ranges from 0.661 to 0.668 with a standard deviation of 0.030 and 0.020, respectively, and 0.030 is the maximum standard deviation with corresponding means of 0.661 and 0.665 (Table 1). As for cough as a first symptom of influenza, the recall ranges from 0.765 to 0.820 with corresponding standard deviations 0.092 and 0.067. The highest standard deviation is 0.110 with a mean of 0.810, and the selectivity ranges from 0.362 to 0.369 with standard deviations of 0.014 and 0.031, respectively, and the maximum standard deviation is 0.031 (Table 2).

The recall in both cases is lower than the selectivity, and this observation indicates that this analysis categorizes patients as infected when they are not, but the high recall indicates that most infected patients did align with the first symptom that we predicted. In the future, we expect to confirm this analysis with data on first symptoms, as opposed to simulated data, but the purpose of this analysis was to display that further study of order of symptoms might lead to earlier recognition.

## Discussion

In this study, we found evidence that supports the notion that there is a most common order of discernible symptoms in COVID-19 that is also different from other prominent respiratory diseases. The most likely initial symptom is fever in the three diseases studied that are caused by coronaviruses (i.e., COVID-19, SARS, and MERS) and cough in influenza. The most likely order of the four easily discernible symptoms is identical in MERS and SARS, but the most likely path of COVID-19 has one key difference. The first two symptoms of COVID-19, SARS, and MERS are fever and cough. However, the upper GI tract (i.e., nausea/vomiting) seems to be affected before the lower GI tract (i.e., diarrhea) in COVID-19, which is the opposite from MERS and SARS. In all diseases, we found that fever and cough occur before nausea/vomiting and diarrhea. When observing the set of seven symptoms including three subjective ones (i.e., sore throat, headache, and myalgia), we found that the initial symptoms of the most likely path are the same as in the most likely path of the four discernible symptoms. Also, in both the four and seven symptoms implementations, the GI tract symptoms are last. A separate MERS dataset included the initial symptoms of patients on admission, which listed the symptoms from highest to lowest probability as fever, myalgia, cough, and diarrhea (18). This order is similar to the most likely path that we determined. A very small percent of patients experienced diarrhea as an initial symptom. This report suggests that diarrhea as an early symptom indicates a more aggressive disease, because each patient in this dataset that initially experienced diarrhea had pneumonia or respiratory failure eventually (Supplemental Table 3). We propose that these patients may be experiencing a more aggressive form of the disease and have accelerated through the most likely path, having already experienced diarrhea. These findings align with another dataset provided for SARS, which also contained the percentage of the various symptoms to be reported first (Supplemental Table 4). The highest reported symptom is fever, followed by cough or dyspnea, and then finally, a small percent of patients reported diarrhea (19). This order confirms the most likely paths that we have determined. The observation that diarrhea was very uncommon as a first symptom and had a non-zero probability of occurrence is consistent with our analysis. This aligns with our hypothesis that early occurrence of diarrhea implies that those patients may have a much more aggressive form of the disease.

The simulation data used to approximate the state and transition probabilities in the Stochastic Progression Model relies on the assumption that symptoms included in the model are independent. Using the definition of independence, we observed the individual probabilities of fever and cough in a dataset from a case study of influenza, and we found that the product of the individual probabilities of fever and cough is almost equal to the probability of both occurring (21). Considering this outcome, we proceeded under the assumption of independence, which we will reevaluate when more symptom data becomes available. We simulated combinations of symptoms for 500,000 patients, which we chose because it was the lowest attempted number that empirically produced the theoretical expected outcome for random frequency symptoms: that all paths would be equally likely, up to 100ths of a decimal place. We then utilized these simulated patients to approximate the state probabilities and transition probabilities described above.

This study supports the idea that symptoms occur in a predictable order, but future work is needed to improve aspects of the Stochastic Progression Model and confirm the results found here. Our finding that COVID-19 first presents with a fever supports the recommended measures by the CDC which state that the public should take their temperature at home and when entering facilities as an early checking method (29). This application of the Stochastic Progression Model may be improved if there were objective ways to measure the more subjective symptoms (i.e., sore throat, headache, and myalgia). Also, improved error calculations of the transition probabilities would lead to more accurate results. Our current error calculation is conservative, because when more symptoms were added, we observed that the error compounded as we progressed further down the paths (Supplemental Figures 2–13). The conservative error estimate creates issues in discerning the difference in probabilities of symptoms. Specifically, in implementations of seven symptoms, the likelihoods are more difficult to ascertain due to subjective reporting and compounding error calculations. Datasets that contain the order of symptoms for each patient would lower the error. Additionally, these sorts of datasets would better the approximations of the transition probabilities and increase accuracy. This improvement could be achieved by physicians implementing the practice of recording the order of occurrence of symptoms. With this information, we may approximate the likelihood of a patient acquiring a symptom based on their current symptoms with patient data instead of simulations based on frequency. Applying objective criteria for symptoms, improving error calculations, and collecting the order of symptoms would not only allow us to improve our findings here, but also allow the Stochastic Progression Model to predict orders of a larger set of symptoms. The optimal form of the Stochastic Progression Model would be developed by determining state probabilities from observed true frequencies of patients' symptoms and determining transition probabilities from the patients' true order of symptoms. However, until this data is available, improved approximations, simulations and error calculations are needed.

Furthermore, when analyzing fever as the first symptom of COVID-19, a low selectivity indicates a high Type I error (i.e., rate of false positive), and a high recall indicates a low Type II error (i.e., rate of false negative). We found a moderate selectivity value and as a result, a moderate Type I error in this case. This Type I error is acceptable in our use of investigating fever as an initial symptom of COVID-19, because it suggests that more people get tested who are not infected, rather than less people get tested who are infected, as with Type II error (30). We are not proposing initial symptom as a diagnostic test, but instead as a possible sign to get tested. COVID-19 outbreaks in clusters, and these unusual clusters of disease are characteristic of a pandemic disease that must be addressed immediately with aggressive testing to curb transmission (31).

The importance of knowing first symptoms is rooted in the need to stop the spread of COVID-19, a disease that is two to three times more transmissible than influenza and results in outbreaks of clusters (3, 4). There is a heightened risk in COVID-19 being passed on, so faster testing and social distancing are important, especially when social distancing and quarantine measures are relaxed. Our results assert that fever is the most likely symptom to occur first in symptomatic adult patients with COVID-19. We hope that the hypotheses generated in this work are tested with prospective clinical data to confirm that a cough occurs first more often in influenza and likewise fever in COVID-19. We believe that early detectors that any individual can recognize to seek medical attention earlier is useful. In addition, datasets that contain information of symptom order and strains of COVID-19 allow for further studies that may determine whether onset of symptoms vary in specific strains (32), and whether risk factors, such as obesity (33), and environmental factors, such as temperature (34) affect symptom order. To slow the spread of COVID-19, our results support the practice that fever should be tested before allowing entry to facilities and that those with fever should immediately seek medical attention for diagnosis and contact tracing. Such measures as these may help to reduce transmission despite the high contagion of SARS-CoV-2.

## Data Availability Statement

Publicly available datasets were analyzed for this study. These can be found here: https://www.who.int/publications-detail/report-of-the-who-china-joint-mission-on-coronavirus- disease-2019-(covid-19), https://www.nejm.org/doi/full/10.1056/NEJMoa2002032, https://jamanetwork.com/journals/jamainternalmedicine/fullarticle/485554, https://onlinelibrary.wiley.com/doi/full/10.1111/resp.13196, https://www.journalofinfection.com/article/S0163-44531630209-2/abstract, https://jamanetwork.com/journals/jama/fullarticle/196681, https://jamanetwork.com/journals/jama/fullarticle/2761044, https://www.cambridge.org/core/journals/infection-control-and-hospital-epidemio logy/article/symptoms-of-influenza-virus-infection-in-hospitalized-patients/8F1B478BA4B861D356393EA77AD8B83B#.

## Author Contributions

JL and JH conceived the model. JL and JM conceived the project. JL created the model. JL, MM, and JM analyzed results. JL and MM wrote the manuscript. PK and JH supervised the project. All authors read, edited, and approved the final manuscript.

## Conflict of Interest

MM is employed by the company Nexus Development PA LLC. JM is employed by the company NanoCarrier Co., Ltd. The remaining authors declare that the research was conducted in the absence of any commercial or financial relationships that could be construed as a potential conflict of interest.
