# Supplementary material for: Modeling the Onset of Symptoms of COVID-19
Source: Front Public Health. 2020 Aug 13;8:473. doi: 10.3389/fpubh.2020.00473 (PMC7438535; doi:10.3389/fpubh.2020.00473)
Supplement: Supplementary file 1 [file Data_Sheet_1.PDF]

# Supplemental Materials

| Datasets used for 4 Symptom Stochastic Progression Models | | | | | | | | |
|---|---|---|---|---|---|---|---|---|
| | Random | COVID-19 with N=55,924[1] | COVID-19 with N=1,099[2] | Severe COVID-19 with N=173[2] | Nonsevere COVID-19 with N=926[2] | Influenza with N=2,470[3] | MERS with N=245[4] | SARS with N=357[4] |
| Fever | 0.500 | 0.879 | 0.887 | 0.881 | 0.919 | 0.680 | 0.841 | 0.997 |
| Cough | 0.500 | 0.677 | 0.678 | 0.673 | 0.705 | 0.930 | 0.633 | 0.583 |
| Nausea/ Vomiting | 0.500 | 0.050 | 0.050 | 0.046 | 0.069 | 0.010* | 0.151 | 0.154 |
| Diarrhea | 0.500 | 0.037 | 0.038 | 0.035 | 0.058 | 0.010* | 0.204 | 0.174 |

*Although adult patients at times may experience vomiting and diarrhea when infected with influenza, these symptoms are rare[5]. Therefore we approximate the frequency of these symptoms as 0.010 in this case.

**Supplemental Table 1 Frequencies of Four Symptoms from Various Clinical Datasets**
Frequencies that were used to simulate patients for the Stochastic Progression Model of discernible symptoms.

| Datasets used for 7 Symptom Stochastic Progression Models | | | | | |
|---|---|---|---|---|---|
| | COVID-19 with N=55,924[1] | COVID-19 with N=1,099[2] | Influenza with N=2,470[3] | MERS with N=245[4] | SARS with N=357[4] |
| Fever | 0.879 | 0.887 | 0.680 | 0.841 | 0.997 |
| Cough | 0.677 | 0.678 | 0.930 | 0.633 | 0.583 |
| Sore Throat | 0.139 | 0.139 | 0.840 | 0.135 | 0.171 |
| Headache | 0.136 | 0.136 | 0.910 | 0.188 | 0.389 |
| Myalgia* | 0.148 | 0.149 | 0.940 | 0.400 | 0.591 |
| Nausea/ Vomiting | 0.050 | 0.050 | 0.010** | 0.151 | 0.154 |
| Diarrhea | 0.037 | 0.038 | 0.010** | 0.204 | 0.174 |

*For the two COVID-19 datasets myalgia is reported as myalgia or arthralgia. We assume most of these cases have myalgia and use it as the frequency for myalgia.

**Although adult patients at times may experience vomiting and diarrhea when infected with influenza, these symptoms are rare[5]. Therefore we approximate the frequency of these symptoms as 0.010 in this case.

**Supplemental Table 2 Frequencies of Seven Symptoms from Various Clinical Datasets**
Frequencies that were used to simulate patients for the Stochastic Progression Model of commonly observed symptoms in respiratory disease.

| Initial Symptoms in MERS[6] | | | | |
|---|---|---|---|---|
| | Without Pneumonia | With Pneumonia | Resipiratory Failure | Overall |
| Fever | 0.182 | 0.714 | 0.778 | 0.559 |
| Cough | 0.308 | 0.316 | 0.385 | 0.333 |
| Myalgia | 0.385 | 0.526 | 0.154 | 0.378 |
| Diarrhea | 0.000 | 0.053 | 0.154 | 0.067 |

**Supplemental Table 3 Initial Symptom Frequency in MERS**
Frequency of symptoms reported by patient as initial in one dataset for MERS, overall and separated by eventual condition of patient.

| | Initial Symptoms in SARS[7] |
|---|---|
| Fever | 0.736 |
| Cough or Dyspnea | 0.354 |
| Diarrhea | 0.063 |

**Supplemental Table 4 Initial Symptom Frequency in SARS**
Frequency of symptoms reported by patient as initial in one dataset for SARS.

| Datasets used for Sensitivity and Specificity Calculations | | | |
|---|---|---|---|
| | COVID-19 with N=138[8] | Influenza with N=20[9] | Negative for Influenza with N=400[9] |
| Fever | 0.986 | 0.450 | 0.183 |
| Cough | 0.594 | 0.750 | 0.298 |
| Nausea/ Vomiting* | 0.101 | 0.010** | 0.010** |
| Diarrhea | 0.101 | 0.010** | 0.010** |

*Nausea and vomiting are reported separately for the COVID-19 dataset here. We assume most people with nausea, frequency of 0.101, have vomiting, frequency of 0.036, and therefore we use nausea's frequency here.

**Although adult patients at times may experience vomiting and diarrhea when infected with influenza, these symptoms are rare[5]. Therefore we approximate the frequency of these symptoms as 0.010 in this case.

**Supplemental Table 5 Frequencies of Four Symptoms from Various Clinical Datasets to Determine Sensitivity and Specificity**
Independent frequencies that were used to simulate patients in order to determine the sensitivity and specificity of first symptoms as early indicators.

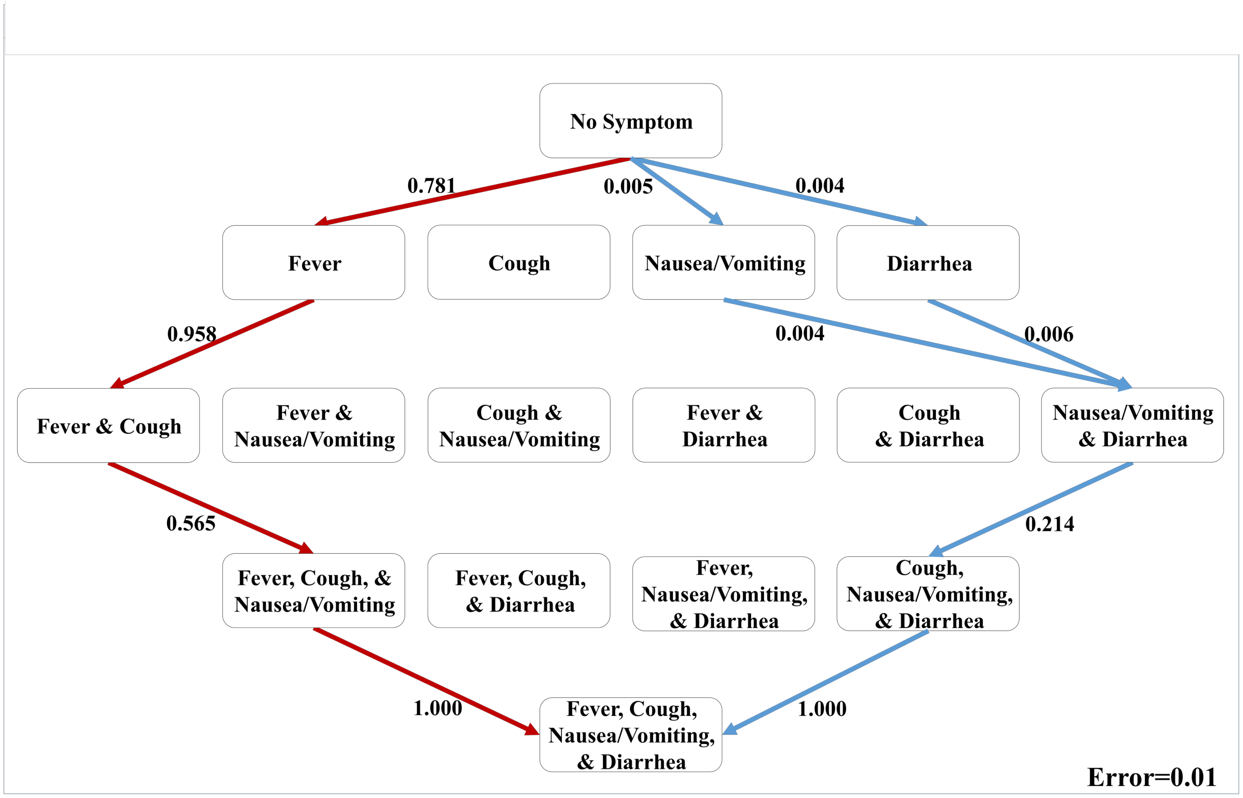


**Supplemental Figure 1 Stochastic Progression Model of Four Symptoms for COVID-19 with N=1,099[2]**

The most (red) and least (blue) likely paths of discernible symptoms for COVID-19 with N=1,099. Used as a confirmation set.

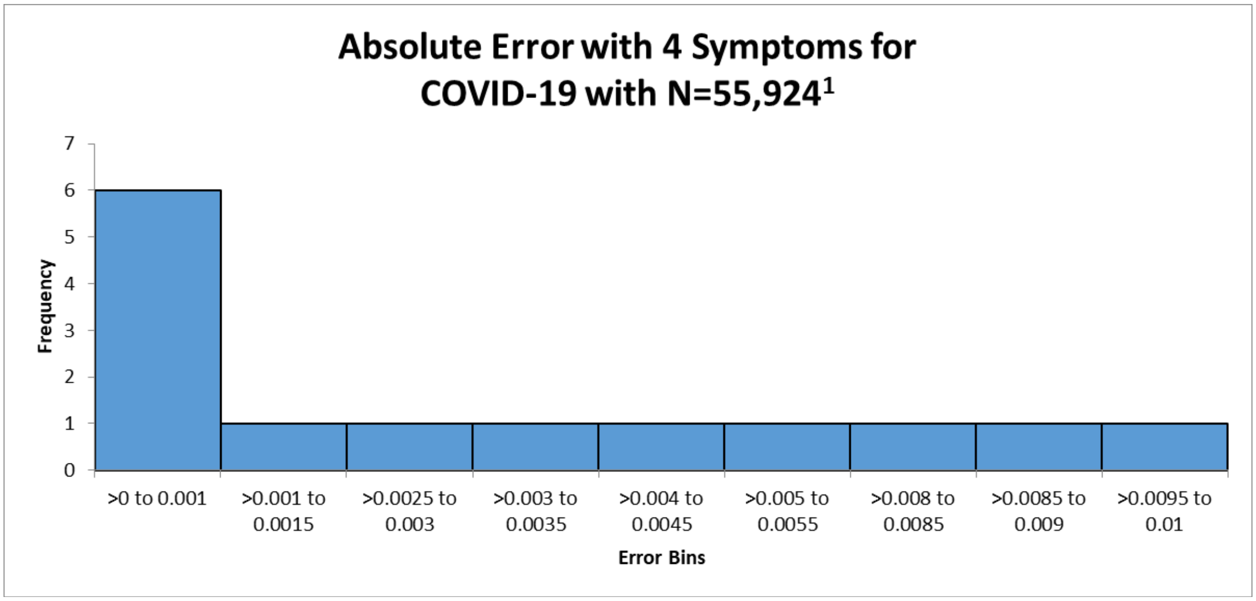


**Supplemental Figure 2 Absolute Error Distribution in COVID-19 with N=55,924 for Discernible Symptoms**
Maximum error of this distribution was used as a conservative value to discern differences in transition probabilities for likely paths.

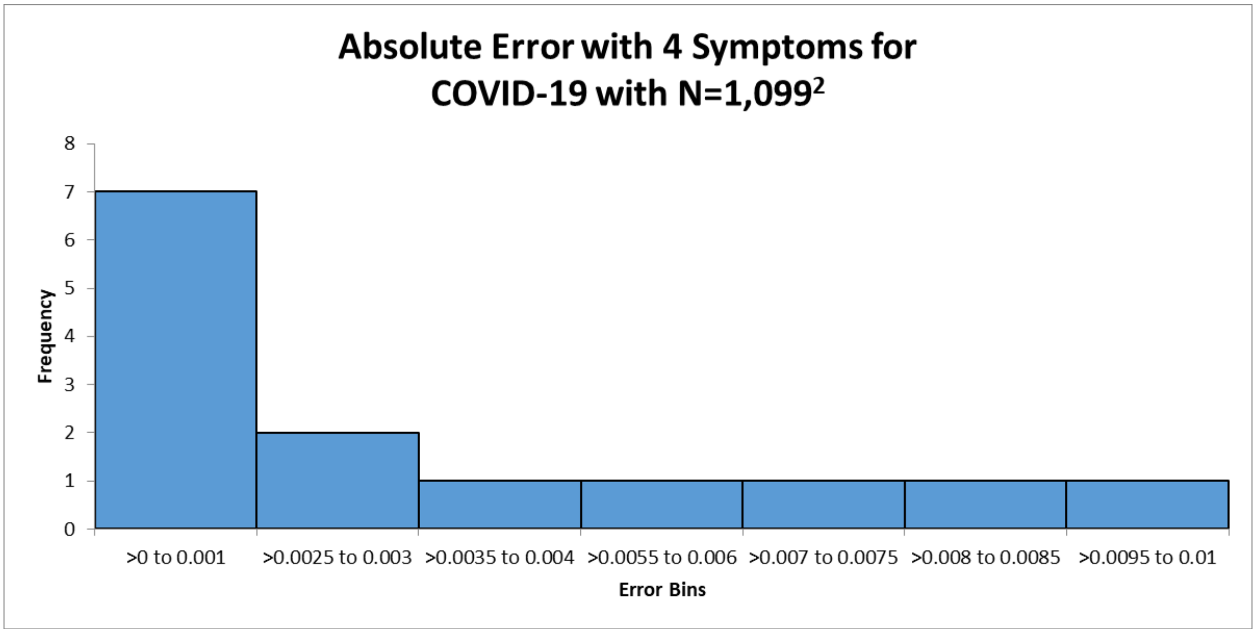


**Supplemental Figure 3 Absolute Error Distribution in COVID-19 with N=1,099 for Discernible Symptoms**
Maximum error of this distribution was used as a conservative value to discern differences in transition probabilities for likely paths.

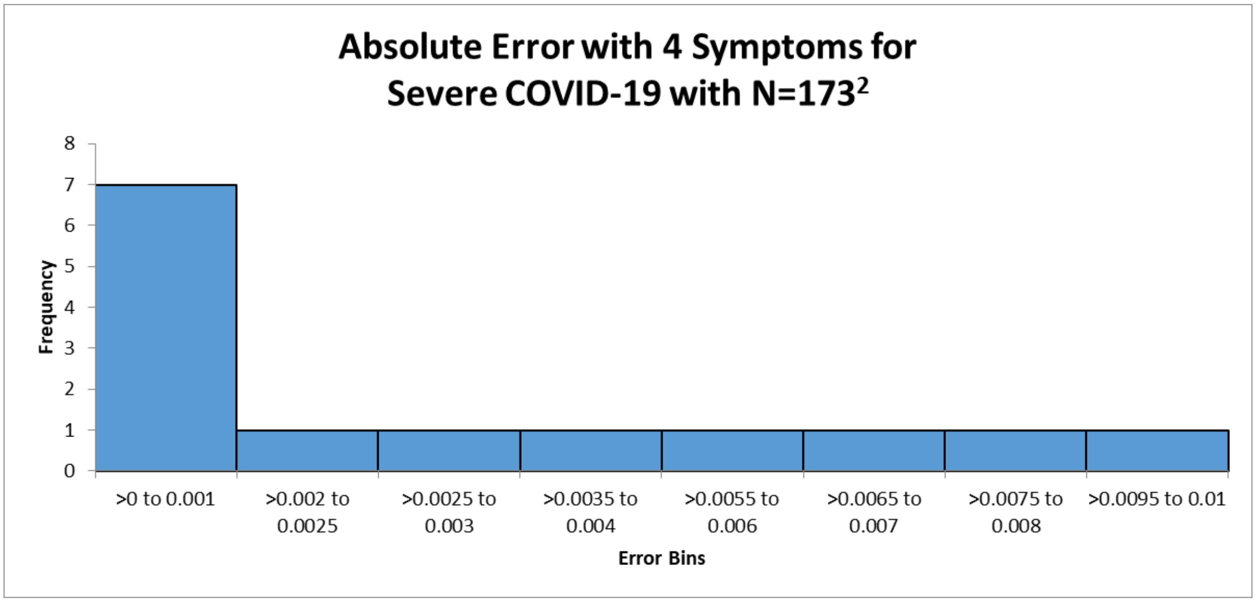


**Supplemental Figure 4 Absolute Error Distribution in Severe COVID-19 with N=173 for Discernible Symptoms**

Maximum error of this distribution was used as a conservative value to discern differences in transition probabilities for likely paths.

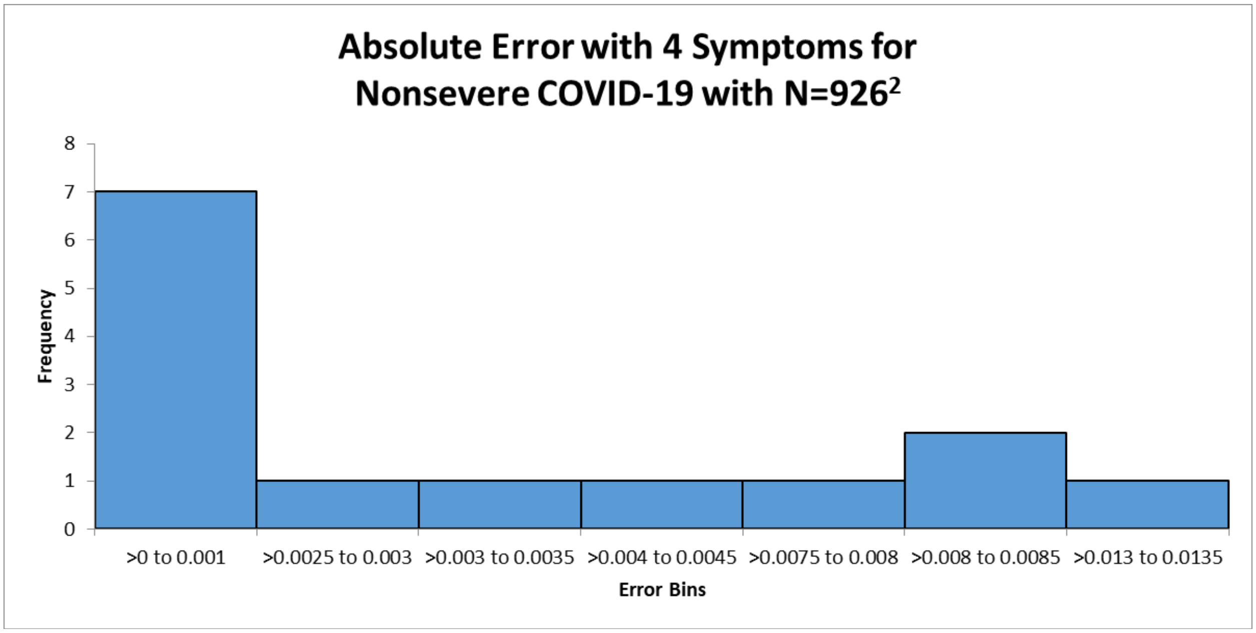


**Supplemental Figure 5 Absolute Error Distribution in Nonsevere COVID-19 with N=926 for Discernible Symptoms**

Maximum error of this distribution was used as a conservative value to discern differences in transition probabilities for likely paths.

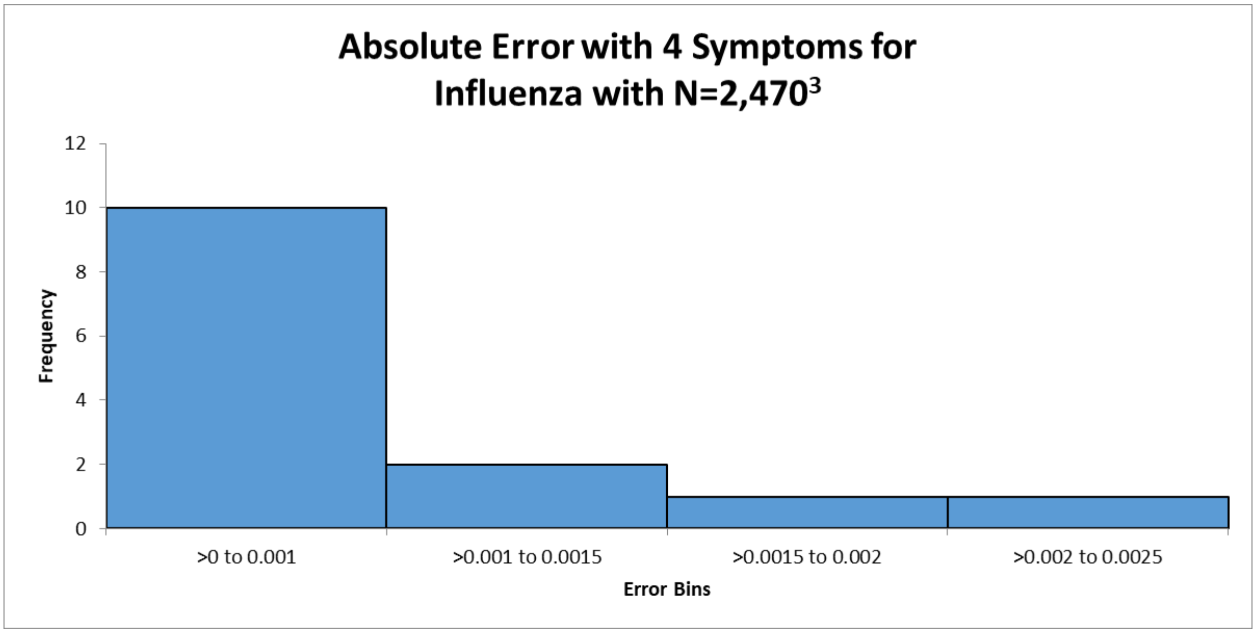


**Supplemental Figure 6 Absolute Error Distribution in Influenza with N=2470 for Discernible Symptoms**
Maximum error of this distribution was used as a conservative value to discern differences in transition probabilities for likely paths.

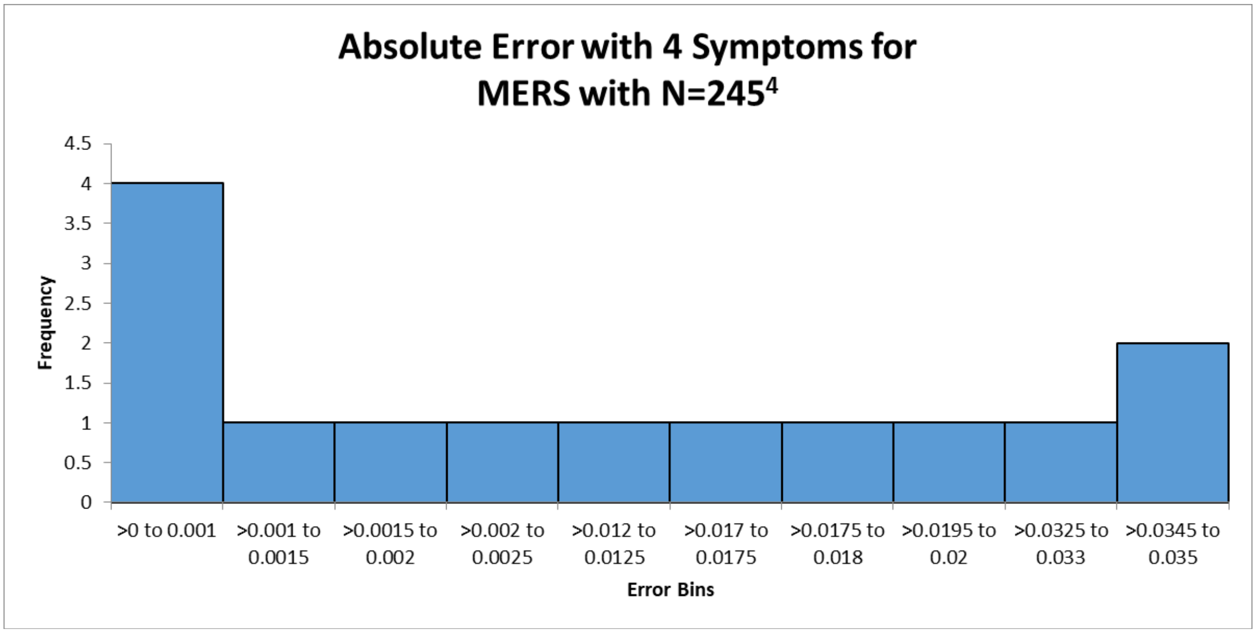


**Supplemental Figure 7 Absolute Error Distribution in MERS with N=245 for Discernible Symptoms**
Maximum error of this distribution was used as a conservative value to discern differences in transition probabilities for likely paths.

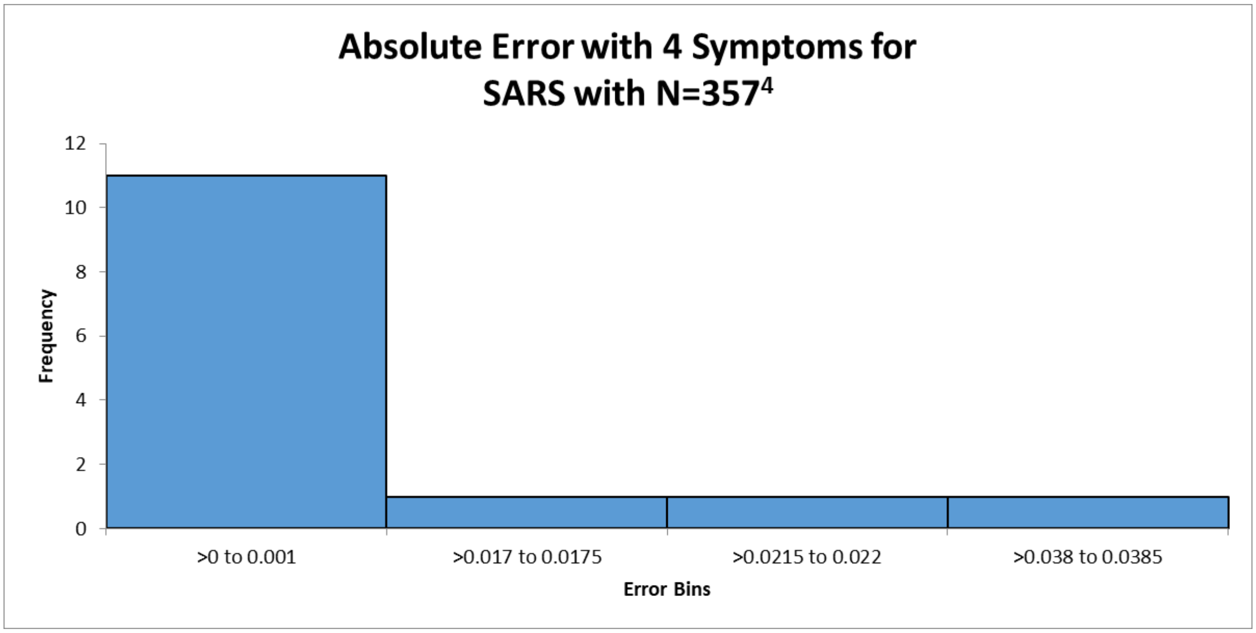


**Supplemental Figure 8 Absolute Error Distribution in SARS with N=357 for Discernible Symptoms**
Maximum error of this distribution was used as a conservative value to discern differences in transition probabilities for likely paths.

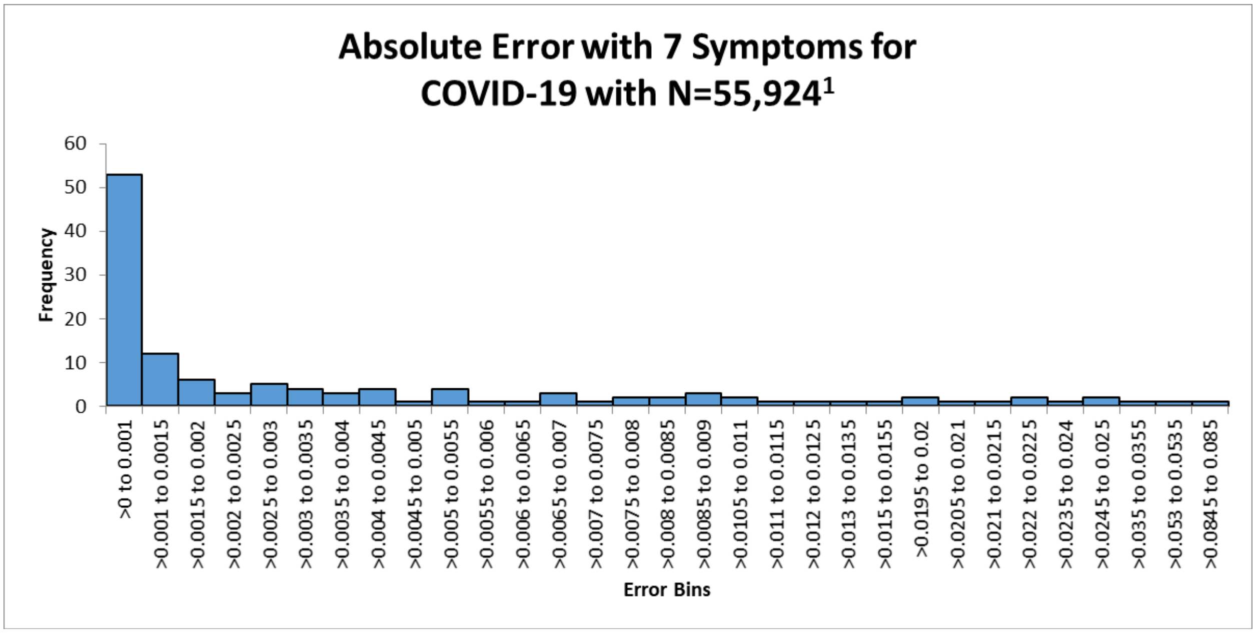


**Supplemental Figure 9 Absolute Error Distribution in COVID-19 with N=55,924 for Common Symptoms in Respiratory Diseases**
Maximum error of this distribution was used as a conservative value to discern differences in transition probabilities for likely paths.

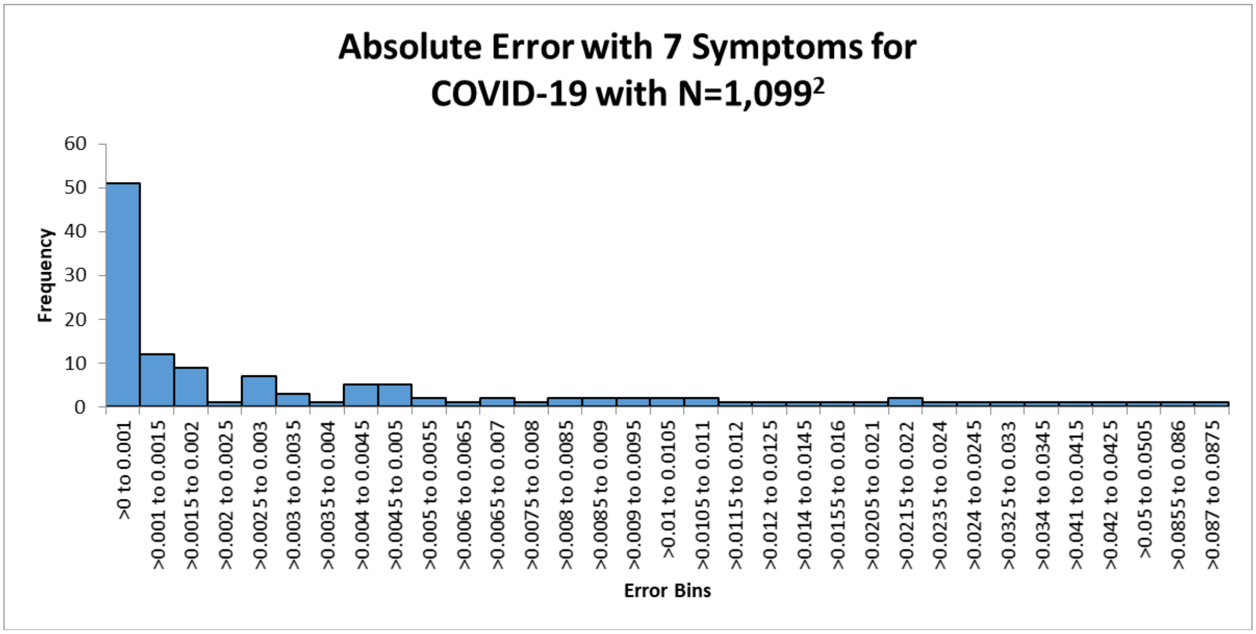


**Supplemental Figure 10 Absolute Error Distribution in COVID-19 with N=1,099 for Common Symptoms in Respiratory Diseases**
Maximum error of this distribution was used as a conservative value to discern differences in transition probabilities for likely paths.

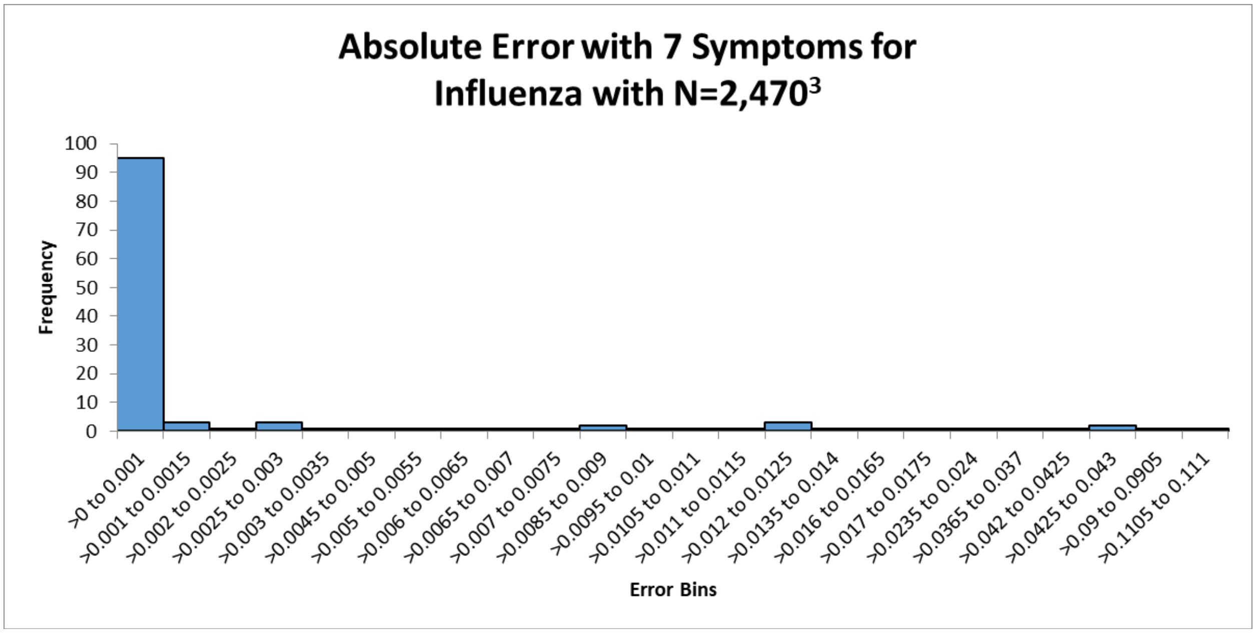


**Supplemental Figure 11 Absolute Error Distribution in Influenza with N=2,470 for Common Symptoms in Respiratory Diseases**
Maximum error of this distribution was used as a conservative value to discern differences in transition probabilities for likely paths.

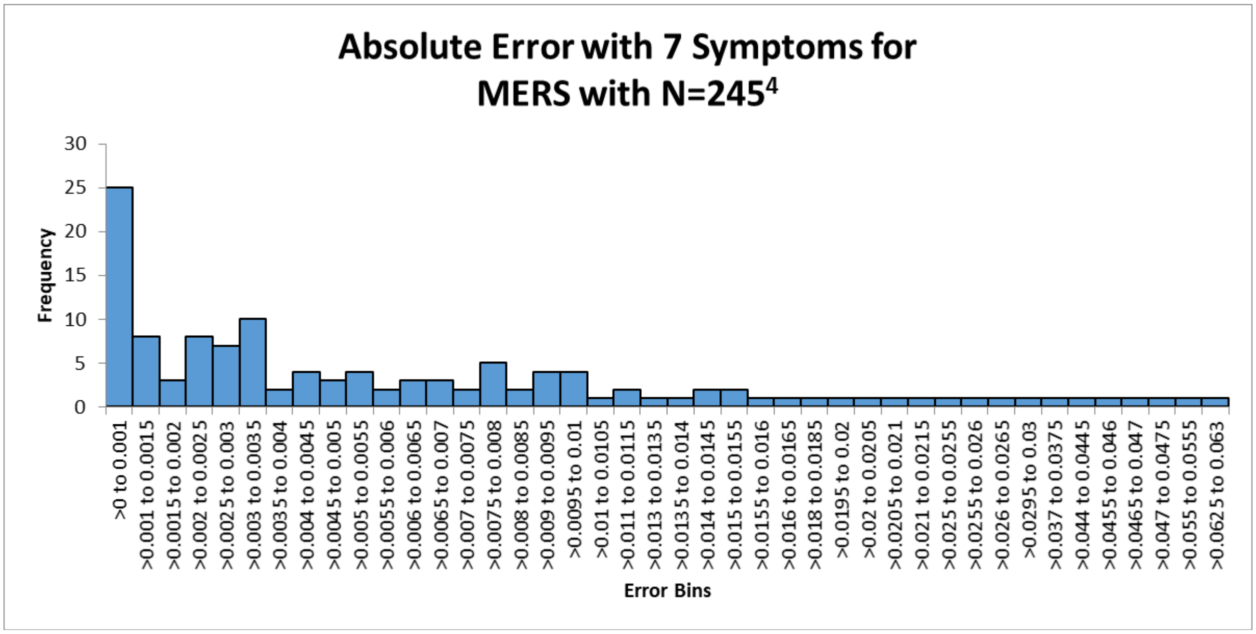


**Supplemental Figure 12 Absolute Error Distribution in MERS with N=245 for Common Symptoms in Respiratory Diseases**
Maximum error of this distribution was used as a conservative value to discern differences in transition probabilities for likely paths.

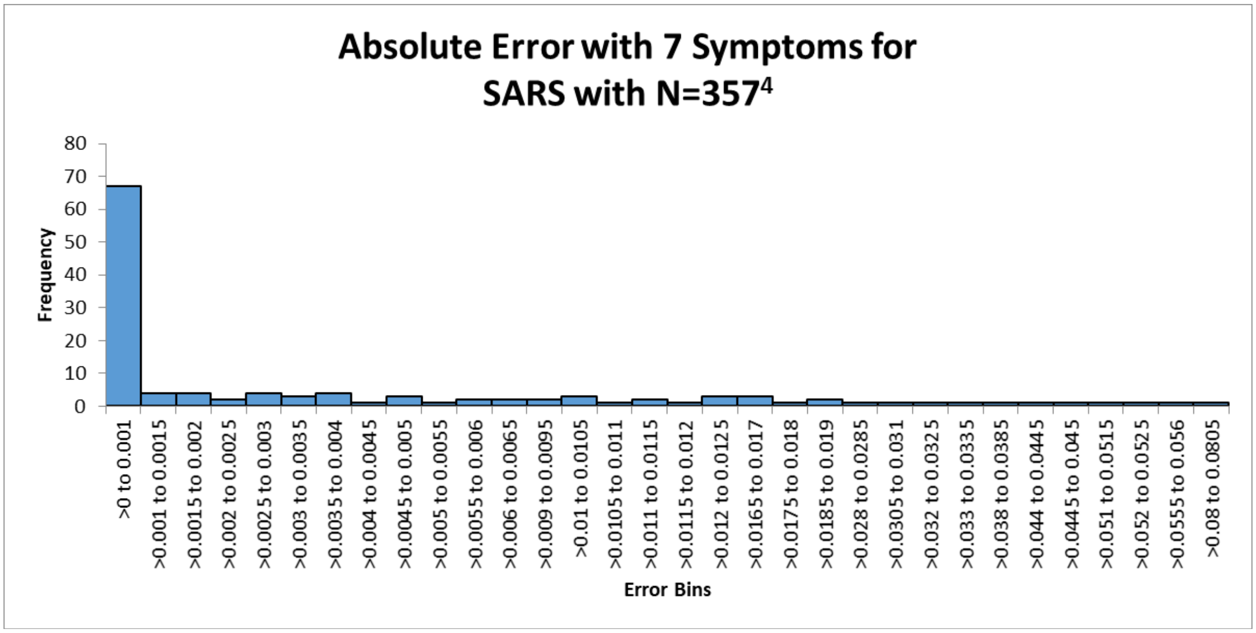


**Supplemental Figure 13 Absolute Error Distribution in SARS with N=357 for Common Symptoms in Respiratory Diseases**
Maximum error of this distribution was used as a conservative value to discern differences in transition probabilities for likely paths.

References

1. Organization, W.H. and W.H. Organization, *Report of the who-china joint mission on coronavirus disease 2019 (covid-19).* Available on-line: https://www.who.int/docs/default-source/coronaviruse/who-china-joint-mission-on-covid-19-final-report. pdf, 2020.
2. Guan, W.-j., et al., *Clinical characteristics of coronavirus disease 2019 in China.* New England Journal of Medicine, 2020.
3. Monto, A.S., et al., *Clinical signs and symptoms predicting influenza infection.* Archives of internal medicine, 2000. **160**(21): p. 3243-3247.
4. Yin, Y. and R.G. Wunderink, *MERS, SARS and other coronaviruses as causes of pneumonia.* Respirology, 2018. **23**(2): p. 130-137.
5. Chan, M.C., et al., *Seasonal influenza A virus in feces of hospitalized adults.* Emerging infectious diseases, 2011. **17**(11): p. 2038.
6. Ko, J.-H., et al., *Predictive factors for pneumonia development and progression to respiratory failure in MERS-CoV infected patients.* Journal of Infection, 2016. **73**(5): p. 468-475.
7. Booth, C.M., et al., *Clinical features and short-term outcomes of 144 patients with SARS in the greater Toronto area.* Jama, 2003. **289**(21): p. 2801-2809.
8. Wang, D., et al., *Clinical characteristics of 138 hospitalized patients with 2019 novel coronavirus–infected pneumonia in Wuhan, China.* Jama, 2020.
9. Van den Dool, C., et al., *Symptoms of influenza virus infection in hospitalized patients.* Infection Control & Hospital Epidemiology, 2008. **29**(4): p. 314-319.
